# Development, optimization, and characterization of polymeric micelles to improve dasatinib oral bioavailability: Hep G2 cell cytotoxicity and in vivo pharmacokinetics for targeted liver cancer therapy

**DOI:** 10.1016/j.heliyon.2024.e39632

**Published:** 2024-10-22

**Authors:** Rehan shaikh, Sankha Bhattacharya, Suprit D. Saoji

**Affiliations:** a Department of Pharmaceutics, School of Pharmacy & Technology Management, SVKM’S NMIMS Deemed-to-be University, Shirpur, Maharashtra, 425405, India; b Department of Pharmaceutical Sciences, Rashtrasant Tukadoji Maharaj Nagpur University Nagpur, Mahatma Jyotiba Fuley Shaikshanik Parisar, University Campus, Amravati Road, Nagpur, 440033, Maharashtra, India

**Keywords:** Dasatinib-loaded micelles, Soluplus® / TPGS, Characterization techniques, Drug entrapment efficiency, In vitro release, Cytotoxicity studies

## Abstract

The efficacy of dasatinib (DAS) in treating hepatocellular carcinoma (HCC) is hindered by its poor bioavailability, limiting its clinical potential. In this study, we explored the use of TPGS-Soluplus micelles as an innovative drug delivery platform to enhance DAS solubility, stability, and therapeutic impact. A series of TPGS-Soluplus copolymers were synthesized, varying the D-α-tocopheryl polyethylene glycol succinate (TPGS) forms (1000, 2000, and 3500) and adjusting the TPGS to Soluplus weight ratios (1:1, 1:2, and 1:3). Our goal was to identify the optimal formulation with the highest entrapment efficiency, smallest particle size, and enhanced drug loading. The TPGS1000-Soluplus copolymer, with a DAS-to-polymer ratio of 1:30 and a TPGS ratio of 1:2, demonstrated superior performance, achieving an entrapment efficiency of 64.479 ± 1.45 % and drug loading of 5.05 ± 1.01 %. The DAS-loaded micelles (DAS-PMs) exhibited a notably small particle size of 64.479 ± 1.45 nm and demonstrated controlled release kinetics, with 85.60 ± 5.4 % of the drug released over 72 h.

Cellular uptake studies using Hep G2 cells revealed significantly enhanced absorption of DAS-PMs compared to free DAS, reflected in lower IC50 values in MTT assays at 24 and 48 h. Pharmacokinetic analysis further highlighted the benefits of the DAS-PMs, with an AUC0-∞ 2.16 times higher and mean residual time (MRT) 1.3 times longer than free DAS, a statistically significant improvement (p < 0.01). These findings suggest that TPGS-Soluplus micelles offer a promising strategy for improving the bioavailability and efficacy of DAS in HCC treatment, presenting a potential new therapeutic avenue for patients with limited options. This innovative formulation could significantly enhance DAS delivery, potentially leading to improved clinical outcomes in liver cancer therapy.

## Highlights

• Ionic Complexation and Nanoprecipitation for DAS-PMs Development.
• Comprehensive Characterization via FT-IR, DSC, XRD, SEM, TEM.
• High Yield Process for DAS-PMs Large-Scale Production.
• DAS-PMs show slow, sustained drug release at pH 7.4 buffer.
• Excellent In-Vitro and In-Vivo Anti-Liver Cancer Effects demonstrated by DAS-PMs.

## Graphical abstract

**Figure:**
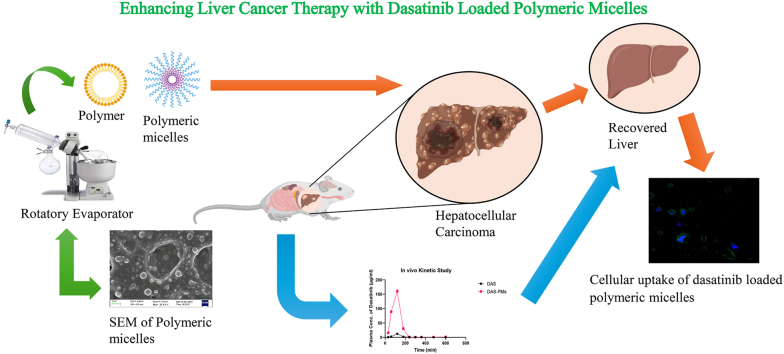

## 1 Introduction

Hepatocellular carcinoma (HCC), a predominant form of primary liver cancer, is increasingly becoming a global health concern. The incidence of HCC is rising at an alarming rate, with projections indicating that cases could surpass one million annually by 2025. Several etiological factors contribute to the development of HCC, including chronic infections with hepatitis B or C viruses, liver cirrhosis, excessive alcohol intake, obesity, diabetes, prolonged use of anabolic steroids, exposure to aflatoxins, and genetic conditions such as hemochromatosis [1,2,3]. A critical challenge in the management of HCC is its typically late diagnosis, which often renders curative treatments like liver transplantation or surgical resection unfeasible. For advanced stages of HCC, systemic therapies remain the primary treatment option. The U.S. Food and Drug Administration (FDA) has approved two main systemic therapies: the multikinase inhibitors sorafenib and lenvatinib. These drugs work by targeting cancerous cells throughout the body and have been shown to improve patient outcomes in advanced HCC cases [4,4,5].Despite their efficacy, sorafenib and lenvatinib are associated with significant adverse effects. Sorafenib, for instance, commonly causes gastrointestinal disturbances, weight loss, and dermatological issues that can lead to discontinuation of treatment. Moreover, resistance to sorafenib frequently develops, limiting its long-term effectiveness.

Recent research has identified dasatinib (DAS) as a promising alternative for HCC treatment. DAS targets crucial molecules involved in tumour progression, such as glypican-3 and sulfatase-2, thereby disrupting cancer-promoting pathways. Additionally, DAS has been shown to reduce the expression of lncRNA-AF08593 and the glycolytic enzyme hexokinase-2, leading to inhibited tumour glycolysis and induced apoptosis in cancer cells [6]. However, DAS poses significant challenges for clinical use due to its poor solubility and low bioavailability. These limitations necessitate innovative delivery methods to enhance DAS's therapeutic efficacy. One promising strategy involves using nanocarriers like micelles. Micelles are self-assembling structures formed from amphiphilic polymers that can encapsulate hydrophobic drugs, thereby improving their solubility and stability. These structures feature a hydrophobic core that solubilizes drugs and a hydrophilic shell that protects them from degradation and interaction with blood components. Among the various micellar formulations, those made with Soluplus and D-α-tocopheryl polyethylene glycol succinate (TPGS) are particularly noteworthy. TPGS, a derivative of vitamin E, enhances drug absorption and bioavailability by inhibiting P-glycoprotein (P-gp), a transporter associated with multidrug resistance. The combination of TPGS and Soluplus in micelles has shown promising results in enhancing the bioavailability and therapeutic efficacy of poorly soluble drugs like DAS. TPGS is also recognized as a safe excipient for oral use by the FDA, further supporting its potential in pharmaceutical applications [7,8,9].

The unique properties of TPGS-Soluplus micelles, including their ability to improve drug encapsulation, stability, and cellular uptake, make them a compelling option for enhancing DAS delivery. These micelles can also offer controlled drug release and targeted delivery to cancer cells, potentially reducing systemic toxicity and improving therapeutic outcomes [10,[11], [12], [13]]. We hypothesize that TPGS-Soluplus copolymer micelles will significantly enhance the solubility, stability, and bioavailability of DAS, thereby improving its therapeutic efficacy against HCC [[14], [15], [16]].To test this hypothesis, we developed DAS-loaded micelles using various TPGS analogues and different molecular weight ratios of TPGS to Soluplus. We characterized the physical and chemical properties of these micelles and evaluated their cytotoxicity and cellular uptake in human liver cancer cells (Hep G2) [17,18,19,20]. The findings from this study could overlay the way for more effective DAS formulations, providing a robust therapeutic option for patients with advanced HCC. Given the escalating incidence of HCC and the limitations of current treatments, developing improved delivery systems for potent drugs like DAS is of paramount importance. This study aims to address the critical issue of DAS's poor bioavailability by leveraging the advanced properties of TPGS-Soluplus micelles. Through this approach, we seek to enhance DAS delivery and efficacy, potentially offering a new lifeline for HCC patients facing limited treatment option.

The field of drug delivery has experienced notable advancements with micelles emerging as highly promising nanocarriers that can enhance the solubility, stability, and bioavailability of hydrophobic drugs. Micelles, particularly those formed from amphiphilic block copolymers like Soluplus® and D-α-Tocopheryl polyethylene glycol 1000 succinate (TPGS), offer significant potential in improving the therapeutic efficacy of drugs with poor solubility profiles. Dasatinib, a tyrosine kinase inhibitor used in treating chronic myeloid leukemia, exhibits pH-dependent solubility, which limits its oral bioavailability and therapeutic efficiency. While previous efforts have addressed these challenges through various formulation strategies, the use of Soluplus® and TPGS to create mixed micelles represents a novel and underexplored approach.

This research seeks to utilize the distinct characteristics of Soluplus®/TPGS mixed micelles (S/T-MM) to increase the oral bioavailability of dasatinib and enhance its specificity towards liver cancer cells. Notable innovations have been made to enhance the solubility and stability of dasatinib over a wide pH range, specifically targeting the solubility challenges related to pH levels. The ∗∗in vivo∗∗ studies on drug movement in the body showed a significant rise in the area under the curve (AUC) for dasatinib when administered through S/T-MM, suggesting improved absorption by the body. The mixed micelles show great potential in targeting liver cancer cells, providing a promising new option for cancer treatment.

Advanced methods like DLS, TEM, DSC, and XRD were used to fully understand the structural and physical properties of the micelles. Moreover, this research adds to the surface and interface science domain by offering fresh perspectives on designing and enhancing polymeric micelles for drug delivery purposes. Soluplus®'s amphiphilic properties, combined with TPGS's solubilizing abilities, lead to an improved interface that boosts encapsulation efficiency and drug stability. The synergy between these two components results in a significant enhancement of the drug's solubility and bioavailability, surpassing the effects of using just one component. Furthermore, the combination of Soluplus® and TPGS in mixed micelles shows promise as a flexible method for transporting various hydrophobic medications, offering a groundbreaking strategy for drug distribution systems.

This research extensively investigates the creation, improvement, and analysis of Dasatinib-loaded polymeric micelles (DAS-PMs) for improved oral bioavailability and targeting of liver cancer cells. The focal points consisted of micelle formulation utilizing TPGS and Soluplus, analysis of particle size, efficiency in encapsulation, and cytotoxicity testing in Hep G2 cells in vitro. Furthermore, we assessed the pharmacokinetics of DAS delivered via micelles, showing prolonged circulation and enhanced bioavailability.

Future studies may concentrate on increasing production for clinical applications and investigating ligand-targeted or stimuli-responsive micelles for improved accuracy in delivering drugs. Moreover, thorough in vivo research is crucial to validate the therapeutic effectiveness of these formulations in the treatment of hepatocellular carcinoma and other types of cancer. This research sets the groundwork for upcoming developments in micellar drug delivery systems.

## 2 Materials

Dasatinib was a generous gift from the MCN Laboratory. BASF of Ludwigshafen, Germany was the source of Soluplus® which has a molecular weight of approximately 1513 g/mol. PMC Isochem in France provided the TPGS 1000. Acetonitrile and methanol were both procured from E. Merck Specialties Private Ltd., located in Mumbai, India. Spectrum Laboratories, Inc., a company based in the USA, supplied the dialysis membrane. The remainder of the chemicals and solvents were all commercially obtained and were either of analytical grade or superior. The HepG2 cell lines were sourced from the NCCS in Pune, India.

## 3 Methods

### 3.1 Preparation of dasatinib-loaded Soluplus®/TPGS mixed micelles

To prepare dasatinib-loaded mixed micelles, the thin-film hydration method was employed. Dasatinib, Soluplus, and D-α-tocopheryl polyethylene glycol succinate (TPGS) were dissolved in 3 mL of anhydrous methanol within a round-bottom flask. The mixture was stirred at room temperature until fully dissolved. A rotary evaporator was then used to evaporate the methanol, forming a thin film. This film was further dried overnight in a vacuum drying oven to eliminate any remaining organic solvent. The dried film was then rehydrated by adding 10 mL of distilled water, followed by stirring for 30 min, resulting in a clear micelle solution. To remove any unencapsulated dasatinib, the solution was centrifuged at 12,000 g for 5 min. The supernatant, containing the dasatinib-loaded mixed micelles, was collected as an opalescent suspension of Soluplus/TPGS mixed micelles. These micelles are poised for application in drug delivery systems [21].

### 3.2 Characterization of micelles

#### 3.2.1 CMC determination

To determine the Critical Micelle Concentration (CMC) using UV–VIS spectroscopy, a KI/I2 stock solution was prepared by dissolving 0.5 g of iodine (I2) and 1 g of potassium iodide (KI) in 50 mL of water. Lyophilized blank S/T-MM samples were then dissolved in water to create various working solutions with polymer concentrations ranging from 0.01 μg/mL to 0.5 mg/mL. Each working solution was mixed with 25 μL of the KI/I2 solution and kept in the dark at room temperature for 12 h. Following this incubation, the absorbance at 323 nm was measured three times for each sample. The absorbance values were then plotted against the polymer concentration. The CMC was identified as the polymer concentration at which there was a noticeable sharp increase in absorbance [22].

#### 3.2.2 Particle size analysis

Utilizing the Zetasizer Nano ZS 90 apparatus from Malvern Instruments, UK, the dynamic light scattering method was employed to assess the particle dimensions of dasatinib-loaded Soluplus®/TPGS mixed micelles. Each specimen was apportioned and diluted in Milli-Q water preceding the analysis.

#### 3.2.3 Entrapment efficiency (%EE)

To determine the encapsulation efficiency of each formulation, the centrifugation technique was utilized. The formulation mixture was transferred into an Eppendorf tube and centrifuged at 12,000 rpm for 10 min. After centrifugation, the supernatant was carefully collected and filtered. The concentration of free drug in this supernatant was then measured using a UV–visible spectrophotometer at a wavelength of 323 nm. The absorbance readings obtained were used to calculate the free drug concentration, employing an equation derived from a previously established calibration curve.

#### 3.2.4 Preparation of stock and working standard solution by UV analysis

To prepare the stock solution, 10 mg of dasatinib (DAS) was dissolved in 10 mL of methanol, creating a 1 mg/mL solution. This stock solution was then further diluted with methanol to achieve concentrations of 2, 4, 6, 8, and 10 μg/mL. UV–visible spectroscopy was employed to determine the maximum absorption wavelength, where the standard solution was scanned against pure water over a range of 200–400 nm. Using these dilutions, absorbance readings at 323 nm were recorded, and a calibration curve was constructed by plotting the absorbance values against the corresponding concentrations. The resulting overlay of the absorption spectra confirmed the calibration [23].

#### 3.2.5 Estimation of DAS by RP-HPLC analysis

To analyse dasatinib (DAS) using high-performance liquid chromatography (HPLC), a C18 column (5 μm, 4.6 × 100 mm; Kromasil) with a guard column of the same phase was utilized in a PerkinElmer Series 200 chromatography system equipped with a UV–visible detector. The optimal mobile phase was a methanol and water mixture in a 70:30 vol ratio. Before use, this solvent mixture was sonicated and filtered through a 0.45 μm membrane filter. It was then pumped through the column at a flow rate of 0.7 mL/min. The column was equilibrated by pumping the mobile phase through it for at least 30 min before sample injection. For each analysis, 20 μL of the sample was injected into the column, maintained at room temperature, with a total run time of 10 min. To prepare the standard DAS solution, 10 mg of DAS was accurately weighed and transferred to a 10 mL volumetric flask. After dissolving in 5 mL of methanol using sonication, methanol was added to reach the 10 mL mark, yielding a 1000 μg/mL DAS solution [24].

#### 3.2.6 In vitro release of dasatinib

To imitate the journey of polymeric micelles through the gastrointestinal tract, an in vitro drug release test was conducted [25,26]. To simulate drug release from polymeric micelles, a release test was conducted using a phosphate buffer solution at pH 7.4 over 12 h, starting at the 0.5-h mark. The pH of human blood and most bodily tissues is roughly 7.4. Performing the release research at this pH allows us to forecast how dasatinib will behave once taken into the bloodstream and dispersed throughout the body. While dasatinib solubility is greatly reduced at pH 7.4, understanding its release properties under these conditions is critical for determining its bioavailability. This information aids in evaluating how much of the drug can reach the systemic circulation for therapeutic activity. Each test was performed in a medium containing 0.5 % w/v Tween 20 to maintain sink conditions. A dialysis bag containing 1 mL of a freshly prepared dispersion of dasatinib-loaded micelles was sealed with clips and placed into a glass container filled with 90 mL of simulated gastric fluid (SGF). This setup was incubated in a water bath set at 37 °C with agitation at 150 rotations per minute. At specified time intervals (0.5, 1, 2, 3, 4, 6, 8, 10, 12, 48, and 72 h), 5 mL samples of the release medium were withdrawn and replaced with fresh medium to maintain volume consistency. The amount of drug released was measured by analyzing the UV absorbance of the samples at 323 nm. A standard curve for Soluplus in each medium was used for the quantification. The percentage of drug released over time was plotted using these data. The experiment was repeated with three different batches of dasatinib-loaded polymeric micelles to ensure reproducibility and accuracy [27].

#### 3.2.7 In vivo pharmacokinetics

Male Wistar rats, each weighing 200 ± 10 g, were obtained from the Experimental Animal Centre at NMIMS University. The study was conducted in accordance with the guidelines approved by the NMIMS University Animal Experimental Ethics Committee (protocol number SPTM/09/2023/IAEC/05). The rats were divided into two groups, each consisting of six animals, and were randomly assigned to receive either free dasatinib (DAS) or dasatinib-loaded Soluplus®/TPGS mixed micelles (DAS-PMs) (Liu et al., 2023) [28]. The total dose of DAS in two group was 5 mg/kg via intravenous. Blood samples (0.4 mL) were collected from the rats via intravenous injection at specified time intervals (0.5, 1, 2, 3, 4, 5, 6, 8, 10, and 12 h) and were immediately transferred into heparinized tubes. The samples were centrifuged at 12,000 rpm for 10 min to separate the plasma, which was then stored at −20 °C until analysis. The plasma samples were subsequently subjected to high-performance liquid chromatography (HPLC) to determine the concentration of dasatinib [29]. The blood extraction process was conducted as follows: Initially, 15 μL of an internal standard solution (methanol, 10 μg/mL) was added to each tube containing 0.4 mL of blood, followed by vortexing for 1 min. Subsequently, 1 mL of acetonitrile was added, and the mixture was vortexed for an additional minute. The samples were then centrifuged at 12,000 rpm for 10 min. After centrifugation, the organic layer was carefully transferred to a sterile tube and evaporated under a gentle nitrogen stream. The dried extracts were reconstituted in 0.4 mL of a methanol/water solution (70/30, v/v) before undergoing high-performance liquid chromatography (HPLC) analysis. The pharmacokinetic parameters were calculated using the DAS 2.1.1 software (Shanghai, China) [29].

#### 3.2.8 Differential scanning calorimetry (DSC)

The structural and physical stability of the samples, including dasatinib (DAS), Dα-tocopheryl polyethylene glycol succinate (TPGS), Soluplus, and DAS-loaded mixed micelles (DAS-PMs), were assessed using Differential Scanning Calorimetry (DSC). The DSC analysis was conducted using a DSC1 differential scanning calorimeter (Mettler Toledo, Schwerzenbach, Switzerland). For each sample, approximately 5 mg was placed in an aluminium pan to form a homogeneous slurry. To each pan, 10 μL of MilliQ water was added to facilitate the measurement. To prevent condensation, the slurry was combined with dry nitrogen. The sealed aluminium pans were heated at a rate of 10 °C/min from 40 °C to 300 °C. The DSC spectra were recorded using the STAR@SW 13.00 software. During the DSC test, key parameters such as the enthalpy of gelatinization (ΔH) and transition temperatures, including onset (To), peak (Tp), and end (Tc) temperatures, were meticulously documented. The enthalpy of gelatinization (ΔH) was determined by integrating the area under the thermogram curve, relative to a baseline, and was expressed in joules per gram of the dried sample. This data provided insights into the thermal behaviour and stability of the compounds and formulations under study [30].

#### 3.2.9 X-ray diffraction (XRD)

X-ray Diffraction (XRD) analysis was utilized to evaluate the crystalline nature of the study components, including pure constituents, physical mixtures, and the optimized formulation. Given that crystalline drugs are often less desirable in pharmaceuticals due to their slow and incomplete dissolution profiles, this analysis is crucial. The Xpert Pro Super X-ray diffractometer from PANalytical (Almelo, Netherlands) was employed for this purpose. Measurements were conducted at room temperature, covering diffraction angles (2θ) from 0° to 40°, to capture key diffraction peaks indicative of the crystalline or amorphous states of the samples. The resulting XRD patterns provided comprehensive insights into the physical state of the drugs and formulations, essential for understanding their dissolution behavior and stability in pharmaceutical applications [31].

#### 3.2.10 Fourier transform infrared spectrometer (FTIR)

An FTIR spectroscopy study was conducted to identify potential physicochemical interactions among the formulation components, elucidate the conjugation of Soluplus and DAS-PMs on the surface of the polymeric micelles, and verify the accuracy of the covalent linkage within the micelles [32]. The Nicolet 5700 FTIR spectrometer (Thermo, Waltham, MA, USA) with a Smart OMNI-sampler attachment was used to record the FTIR spectra of DAS, Soluplus, TPGS, and Qu-PMs. Samples were placed on the plates, and spectra were collected in the 400–4000 cm^−1^ region at a resolution of 1 cm^−1^ [31].

#### 3.2.11 Raman spectroscopy

Raman spectroscopy, a non-destructive analytical technique, provides detailed insights into molecular interactions, phase and polymorphy, crystallinity, and chemical structure by leveraging the interaction of light with the chemical bonds in a material. In this study, LabSpec 6 from Horiba Scientific was used to perform Raman spectroscopy analysis on both placebo and dasatinib-loaded polymeric micelles [33].

#### 3.2.12 Morphological study of polymeric micelles using FESEM & TEM

The JSM-IT800 Field Emission Scanning Electron Microscope (Tokyo, Japan) was used to examine the optimized DAS-PMs. Samples were mounted on metal plates, coated with a gold-palladium mixture to a thickness of 100 Å, and subjected to an accelerated voltage of 20 kV. Additionally, the morphology of the optimized DAS-PMs was visualized using transmission electron microscopy (TEM) with a Hitachi 7500 (Tokyo, Japan), as established by Quan et al. For TEM analysis, carbon-coated polymeric micelles were placed on a copper grid stained with 1 % phosphotungstic acid. The resulting images were analyzed with software that generates digital micrographs [34].

### 3.3 In-vitro cytotoxicity studies

#### 3.3.1 MTT/SRB assay with Flow cytometry

The MTT assay was performed to assess the cytotoxicity of the samples on the HepG2 cell line. Approximately 8000 cells per well were cultured in a 96-well plate for 24 h at 37 °C with 5 % CO2 in DMEM medium supplemented with 10 % FBS and 1 % antibiotic solution. The following day, cells were exposed to various dosages and concentrations ranging from 1 to 2500 μM, prepared using incomplete medium to achieve the desired concentrations [35]. After a 24-h incubation period, 100 μL of 10 % trichloroacetic acid (TCA) was added to each well and incubated for 1 h. Then, the plate was washed with distilled water and allowed to dry at room temperature [36,37]. MTT solution was added to each well at a final concentration of 0.04 % and was allowed to sit for 1 h. After an hour of incubation, the plate was air-dried at room temperature before being cleaned with 1 % (v/v) acetic acid to remove any remaining color. To solubilize the protein-bound dye, Tris base solution (pH = 10.5) was added to a well and shaken for 10 min on an orbital shaker. The protein-bound dye was then detected at 510 nm using an ELISA plate reader (iMark, Biorad, USA) [38].

#### 3.3.2 Cell cycle analysis with Flow cytometry - HepG2

Specimens were placed on cells and incubated for 24 h. The cells that were not subjected to any treatment were the control cells. After the set incubation duration, trypsin was applied to detach the cells. Subsequently, the cells were gathered in a 1.5-mL vial and rinsed with 500 μL of chilled PBS [39]. A suspension of approximately 10^6^ cells was prepared by gently vortexing 100 μL of PBS, resulting in a monodispersed cell suspension with minimal aggregation. The cells were then fixed by adding this solution to centrifuge tubes containing 900 μL of 70 % ethanol on ice, followed by incubation at 4 °C for at least 2 h. Cells can be stored in 70 % ethanol at 4 °C for several weeks. According to Sun et al. after centrifugation post-fixation, the cell pellet was resuspended in 500 μL of Propidium Iodide (PI) staining solution (0.1 % (v/v) Triton X-100, 10 μg/mL PI, and 100 μg/mL DNase-free RNaseA in PBS). The mixture was incubated in the dark for 30 min at room temperature or 10 min at 37 °C. Finally, samples were analyzed using a flow cytometer [40].

#### 3.3.3 Cellular apoptosis with Flow cytometry – HepG2

HepG2 cell lines, obtained from NCCS Pune, were cultured and treated with the compound's IC-50 dosage. Post-treatment, the cells were divided into five groups: unstained cells, control group, annexin-only, PI-only, and treatment. The cells were then resuspended in 1X binding buffer at a concentration of 1 × 10^6^ cells/mL. FITC and PI from Annexin V were added to the appropriately labeled tubes. According to Zhan et al., 1X binding buffer was added to each tube, and they were incubated for 15 min at room temperature. The samples were analyzed using a BD FACS Lyric™ flow cytometer within an hour [41].

#### 3.3.4 In vitro cytotoxicity MTT assay – HepG2

The cytotoxicity of the samples was evaluated using the HepG2 cell line, obtained from NCCS Pune, through the MTT assay. A 96-well plate containing 10,000 cells per well was incubated at 37 °C with 5 % CO2 for 24 h in DMEM medium (Dulbecco's Modified Eagle Medium-AT149-1L) supplemented with 10 % FBS (Foetal Bovine Serum-HIMEDIA-RM 10432) and 1 % antibiotic solution. The following day, the cells were treated with varying concentrations of the samples, while the control cells remained untreated [42]. After a 24-h incubation period, the cell culture was treated with MTT solution and incubated for a further 2 h [43]. After completing the experiment, the culture supernatant was discarded, and the cell layer was dissolved in 100 μL of Dimethyl Sulfoxide (catalog number 67685). The results were measured at 540 nm and 660 nm using a Bio-Rad ELISA plate reader (USA). The IC50 value was calculated using GraphPad Prism 9 software. Images were captured with an AmScope Aptima CMOS digital camera (10 megapixels) under an Olympus CK2 inverted microscope.

#### 3.3.5 ROS estimation with Flow cytometry – HepG2

Cells were seeded into six-well plates at densities ranging from 5000 to 10,000 HepG2 cells per well, using 1 mL of DMEM media supplemented with 10 % FBS and 1 % antibiotic solution. The plates were then incubated at 37 °C with 5 % CO2 for 24 h. Prior to treatment, the old culture medium was replaced with fresh medium, designating untreated cells as controls. Following the protocol by Gupta et al. cells were treated with various agents and further incubated for 24 h. After incubation, the medium was removed, and cells were detached using trypsin-EDTA, transferred to 1.5 mL tubes, and washed with 500 μl of cold PBS. The cell pellet was resuspended in 100 μl of PBS containing 2 μM DCFDA, and samples were incubated for 1 h before analysis using a BD FACS Calibur flow cytometer (USA). Data were processed using version 2.5.1 of the Flowing software program [44].

#### 3.3.6 MMP estimation with Flow cytometry – HepG2

HepG2 cells were cultured, treated with the specified dosage, and incubated for 24 h. Following the incubation, the culture medium was aspirated, and the cells were detached using trypsin-EDTA, transferred to 1.5 mL tubes, and washed with 500 μl of cold PBS. The cell pellet was then resuspended in 400 μl of JC1 staining solution containing 2 μM JC-1 dye. After incubating for 1 h, samples were analyzed using a flow cytometer (BD FACS Canto II) [45].

## 4 Statistical analysis

At least three replicas of every experiment were conducted, and GraphPad Prism 9 (GraphPad Software, Inc., La Jolla, CA) was utilized for analysis. The calibration curve was constructed through linear regression analysis, with the correlation coefficient (R^2^) used to evaluate the fit of the model. Solubility data were examined using one-way ANOVA, and Tukey's post-hoc test was employed to identify significant differences in solubility across various pH levels, with statistical significance set at p < 0.05. In vivo pharmacokinetic parameters, including AUC and Cmax, were determined using non-compartmental analysis, and comparisons between drug formulations were assessed using paired t-tests. Size distribution data were analyzed for mean diameters and polydispersity indices, with statistical significance in size variations between formulations established through ANOVA. IC50 values for different treatments were derived from dose-response curves, and differences in cytotoxicity were evaluated using one-way ANOVA with subsequent post-hoc comparisons. ROS levels were analyzed across groups using two-way ANOVA to explore the effects of different treatments and time points. Additionally, particle size from TEM images was statistically analyzed for mean diameters and variation using standard deviation. In vitro release profiles were assessed through regression analysis to understand release kinetics, and differences in release rates were compared using ANOVA [46].

## 5 Results

### 5.1 Particle size

DAS-loaded polymeric micelles were developed to enhance cellular uptake and target liver cancer. The micelles underwent characterization for particle size and entrapment efficiency. Thirteen formulations of DAS-PMs were prepared and analyzed for their particle sizes, ranging from 52.43 ± 2.12 nm to 131.5 ± 3.12 nm. The optimized formulation exhibited a particle size of approximately 74.477 nm. This ideal batch size was determined to be more effective in reducing cancer activity, highlighting its potential in targeted therapy for liver cancer [47].

### 5.2 Entrapment efficiency (EE%)

Thirteen distinct formulations of DAS-PM polymeric micelles were assessed, showing encapsulation efficiencies ranging from 53 % to 78 %. Understanding that the polymer characteristics significantly impact drug encapsulation efficacy is crucial in this evaluation The optimal formulation was chosen based on achieving the highest entrapment efficiency possible, which measured 78.7 %. Following calculations, it was determined that the final entrapment efficiency of the optimized formulation reached 64.479 % [48].

### 5.3 Characterization of micelles

**Fig. 1:**
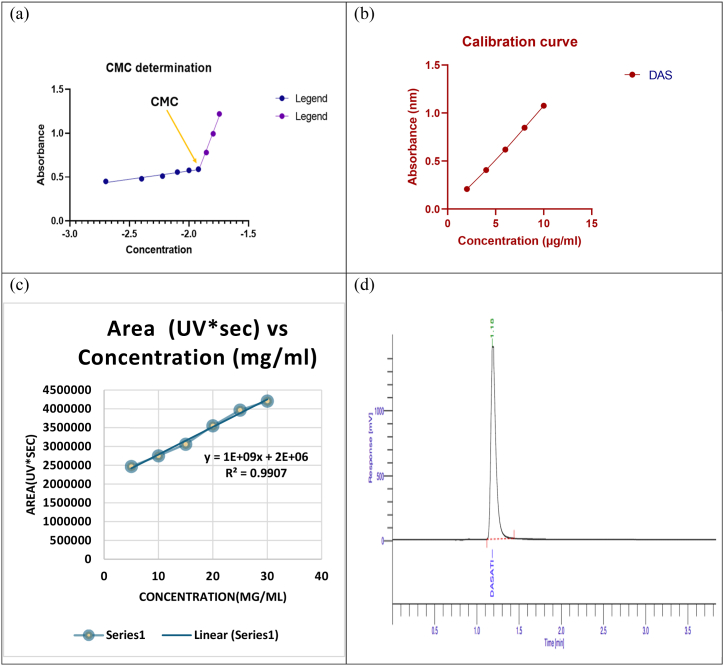
(a)Determination of critical micelle concentration (CMC) for dasatinib-loaded TPGS/Soluplus® mixed micelles**,** (b)UV calibration curve of dasatinib**,** (c)HPLC calibration curve for quantification of dasatinib**,** (d)HPLC calibration curve for quantification of dasatinib.

The critical micelle concentration (CMC) serves as a vital indicator of micellar stability and the ability to form micelles; a lower CMC suggests easier preparation and greater stability of the micelles. Various techniques, such as light scattering, nuclear magnetic resonance (NMR) spectroscopy, conductometry, fluorimetry, calorimetry, and tensiometry, are currently employed to determine the CMC. In the study by Rastogi et al. UV spectrometry was used to determine the CMC of mixed micelles, generating scatter plots depicted in Fig. 1 (a) and plotting linear curves. The intersection of these curves pinpointed the CMC value of the mixed micelles at 4.77 × 10^−2^ mg/mL, which exceeds the manufacturer's reported CMC value for pure Soluplus® (7.6 × 10^−3^ mg/mL), although the manufacturer did not specify the conditions. Incorporating TPGS, which has a higher CMC value of 0.2 mg/mL, likely contributed to this higher CMC. The addition of TPGS adversely affects the self-aggregation of Soluplus® within the micelles. However, different methods and conditions used to calculate the CMC can lead to varying results; for instance, Soluplus® was found to have a CMC of approximately 0.8 mg/mL at 25 °C using isothermal titration calorimetry. Another study indicated a CMC value of approximately 0.016 mg/mL for mixed micelles with a molar ratio of 6:1 (Soluplus®/TPGS). Nevertheless, a CMC below 135 mg/L is considered sufficient to prevent micelle dissociation upon dilution after oral delivery, and our investigation suggests that the mixed micelles can maintain their integrity upon dilution.

### 5.4 Preparation of calibration curve

A calibration curve for dasatinib was generated using a concentration range of 2–10 mg/mL. Standard solutions of dasatinib were prepared by combining methanol and water in 10 mL volumetric flasks at volumes of 2, 4, 6, 8, and 10 mL, each containing a precisely measured amount of dasatinib. The absorbances corresponding to these solutions were plotted on the Y-axis against dasatinib concentrations on the X-axis to construct the calibration curve. Additionally, the overlay spectrum of dasatinib, including the calibration curve, is depicted in Fig. 1(b).

### 5.5 HPLC analysis of DAS

A chromatogram was generated by dissolving 10 mg of DAS in 10 mL of a mobile phase consisting of methanol and water in a 70:30 v/v ratio. The retention time of DAS was determined to be 1.18 min (Fig. 1(c)). The linearity of the DAS method was assessed over a concentration range of 1–6 mg/mL. Calibration graphs were constructed by plotting the peak area of the analyte against the drug concentration in mg/ml. These graphs exhibited linear relationships during calibration, as depicted in Fig. 1(d), where x represents concentration and y represents area. The correlation coefficient (R^2^) for the calibration curve was determined to be 0.9907 (see Table 1).

### 5.6 Experimental design

Table 2 of Design Expert software (DoE) was utilized to investigate and understand the influence of process and content characteristics on quality attributes. Central composite design was employed to modify the experimental design. The primary objective of the study was to identify various independent and dependent factors impacting the formulation of polymeric micelles. Key factors influencing the entrapment efficiency and particle size of polymeric micelles included Soluplus and TPGS. Based on optimization surface plots, specific target values for particle size and entrapment efficiency were selected for further characterization studies. Thirteen different formulations of dasatinib-loaded polymeric micelles were generated using the central composite design. Subsequently, the particle size and entrapment efficiency of each formulation batch were evaluated individually.

**Table 1:** Data used to optimize the formulation for desired characteristics like particle size and entrapment efficiency.

Parameters	Unit	High level	Low level
Soluplus	Mg	160	80
TPGS	Mg	80	20

Factors										
Factor	Name	Units	Type	Sub Type	Minimum	Maximum	Coded Low	Coded High	Mean	Std. Dev.
A	Soluplus	mg	Numeric	Continuous	63.43	176.57	−1 ↔ 80.00	+1 ↔ 160.00	120.00	32.66
B	TPGS	mg	Numeric	Continuous	7.57	92.43	−1 ↔ 20.00	+1 ↔ 80.00	50.00	24.49

Responses								
Response	Name	Units	Observations	Minimum	Maximum	Mean	Std. Dev.	Ratio
R1	Mean particle size	nm	13.00	74.3	80.98	76.65	1.89	1.09
R2	% DEE	%	13.00	63.87	67	65.02	0.9101	1.05

**Table 2:** Design of Experiments (DOE) model (Central Composite) for Dasatinib loaded polymeric micelles formulation.

Std	Run	Factor 1	Factor 2	Response 1	Response 2
		A:Soluplus (mg)	B:TPGS (mg)	Particle size	Entrapment efficiency
		%	%	nm	%
**13**	1	120	50	57.69 ± 2.45	61.2 ± 1.23
**10**	2	120	50	126.46 ± 4.67	75.5 ± 1.12
**7**	3	120	7.57359	69.99 ± 2.28	72.8 ± 1.21
**6**	4	176.569	50	69.30 ± 2.56	73.6 ± 1.34
**8**	5	120	92.4264	67.95 ± 2.48	57.3 ± 1.14
**5**	6	63.4315	50	80.98 ± 3.25	67.2 ± 1.44
**12**	7	120	50	54.92 ± 2.23	72.2 ± 1.22
**2**	8	160	20	72.76 ± 2.49	62.7 ± 0.99
**9**	9	120	50	52.43 ± 2.12	68.4 ± 1.15
**11**	10	120	50	77.95 ± 2.74	71.2 ± 1.7
**1**	11	80	20	60.46 ± 2.31	53.4 ± 1.15
**3**	12	80	80	74.41 ± 2.41	76.1 ± 1.91
**4**	13	160	80	131.5 ± 3.12	78.7 ± 1.65

DOE model (Central Composite) for Dasatinib loaded polymeric micelles formulation. N = 3(mean ± SD).

#### 5.6.1 ANOVA and response analysis for particle size (Quadratic model)

**Fig. 2:**
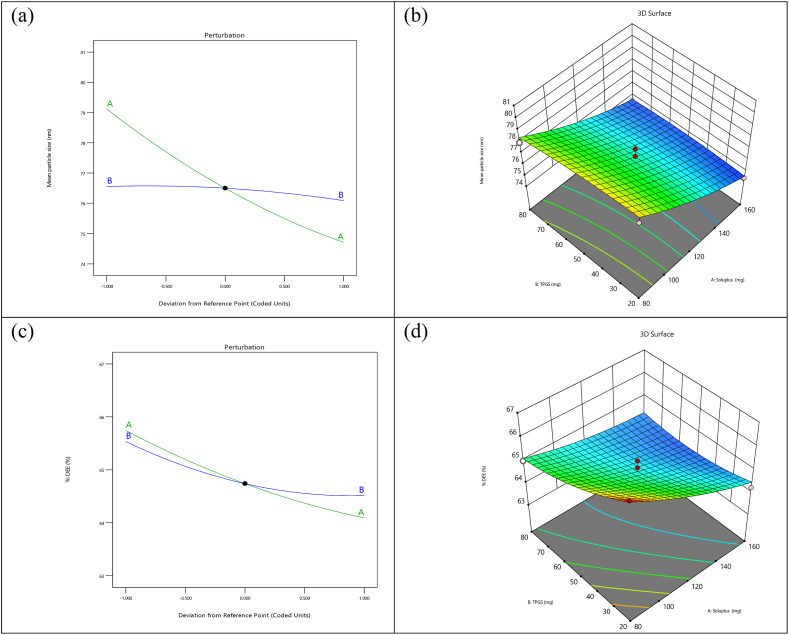
(a-b)Representation of particle size, (c-d)Representation of entrapment efficiency.

The F-value of 34.11 for the model indicates significant results. Such a high F-value can only be attributed to noise with a probability of 0.01 %. In this scenario, A is a crucial model term. Generally, model terms with P-values below 0.0500 are considered significant. Conversely, if the value surpasses 0.1000, the model terms are deemed insignificant. If several unnecessary model terms exist, excluding those required to maintain hierarchy, model reduction could potentially enhance the model. Tables 3 1.98 Lack of Fit F-value implies that there's a 25.90 % probability that noise could produce a considerable Lack of Fit F-value. A slight misfit is permissible for the model to fit appropriately. There is a satisfactory consensus between the variance of the modified R^2^ of 0.9324 and the anticipated R^2^ of 0.8076. Adequate Precision, which is used to quantify the signal-to-noise ratio, should ideally exceed 4. A ratio of 18.663 suggests a robust signal. The model, as depicted in Fig. 2(a and b), can be employed to investigate the design arena. Equation (1) denotes the polynomial equation associated with this model (see Table5).

(Eq.1) Final Equation in Terms of Coded Factors for Particle size = +76.50–2.20 A -0.2332 B + 0.2925 AB +0.4160 A^2^ -0.1765 B^2^

**Table 3:** Quadratic model: Analysis of Variance (ANOVA) Results for Particle Size Distribution Across Experimental Conditions.

Source	Sum of Squares	df	Mean Square	F-value	p-value	
**Model**	41.12	5	8.22	34.11	<0.0001	significant
A-Soluplus	38.76	1	38.76	160.76	<0.0001	
B-TPGS	0.435	1	0.435	1.80	0.221	
AB	0.342	1	0.342	1.42	0.272	
A^2^	1.20	1	1.20	4.99	0.060	
B^2^	0.216	1	0.216	0.898	0.374	
**Residual**	1.69	7	0.241			
Lack of Fit	1.01	3	0.336	1.98	0.259	not significant
Pure Error	0.678	4	0.169			
**Cor Total**	42.80	12

#### 5.6.2 Response 2: entrapment efficacy (Quadratic model) entrapment efficiency analysis due to the effect of Variables

The model's F-value of 12.25 suggests that it might be significant. An F-value this high would only be observed 0.24 % of the time due to random noise. Terms in the model with a P-value of less than 0.0500 are considered significant, in this case, A and B are significant. If the P-value is greater than 0.1000, the terms are not significant. If the model has a large number of unnecessary terms, model reduction could potentially improve it. The F-value of 1.21 for lack of fit indicates that pure error is more relevant than lack of fit. In Table 4, there is a 41.31 % chance that noise will lead to a notable deficiency in the fit F-value. It is advantageous to have an insignificant lack of fit in the model because we desire it to be fitting. There is a discrepancy of less than 0.2 between the adjusted R^2^ of 0.8241 and the predicted R^2^ of 0.5686, which shows a reasonably good consensus. Adeq Precision evaluates the signal-to-noise ratio. Ideally, the ratio should exceed 4. A ratio of 11.648 indicates that the signal is adequately strong. This model can be employed, as depicted in Fig. 2(c and d), to navigate the design space. Equation (2) represents the polynomial equation that was derived for this model. Final Equation in Terms of Coded Factors

(Eq.2) EE = +64.74–0.8251 A -0.5107 B + 0.4100 AB +0.1791 A^2^ +0.2866 B^2^

**Table 4:** ANOVA Analysis: Evaluation of Entrapment Efficiency Response Across Formulation. Variants Response 2: Entrapment efficiency.

Source	Sum of Squares	df	Mean Square	F-value	p-value	
**Model**	8.92	5	1.78	12.25	0.0024	significant
A-Soluplus	5.45	1	5.45	37.39	0.0005	
B-TPGS	2.09	1	2.09	14.32	0.0069	
AB	0.672	1	0.672	4.62	0.068	
A^2^	0.223	1	0.223	1.53	0.2557	
B^2^	0.571	1	0.571	3.92	0.0881	
**Residual**	1.02	7	0.145			
Lack of Fit	0.485	3	0.161	1.21	0.4131	not significant
Pure Error	0.534	4	0.133			
**Cor Total**	9.94	12

**Table 5:** Characteristics of the Optimized Formulation after iterative. The optimized formulation after the calculation is as follows.

Factor	Optimum value
Soluplus	160 mg
TPGS	20 mg

### 5.7 Differential scanning calorimetry (DSC)

**Fig. 3:**
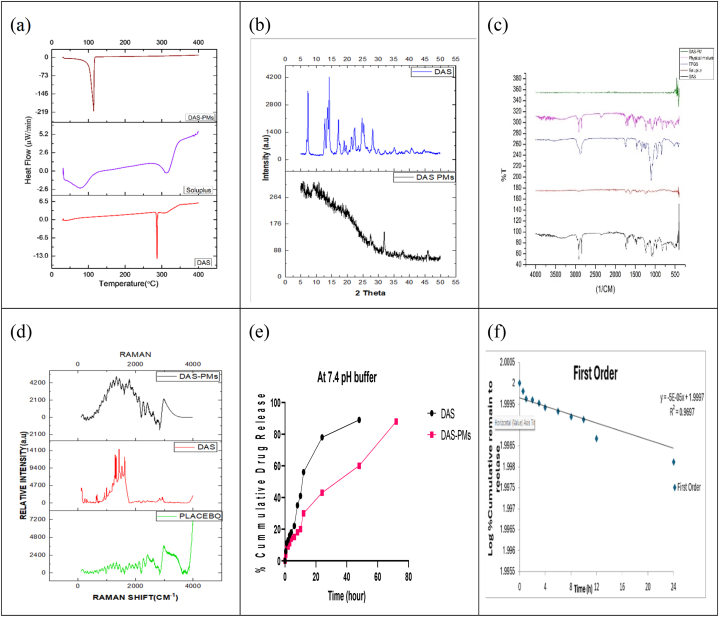
(a)Differential scanning calorimetry (DSC) curves of dasatinib (DAS), Soluplus®, and the dasatinib-loaded Soluplus®/TPGS mixed micelles, (b) Powder X-ray diffraction (XRD) curves of dasatinib (DAS), and the dasatinib-loaded Soluplus®/TPGS mixed micelles, (c) FTIR of DAS (i), Soluplus (ii), TPGS (iii), physical mixture (iv), DAS-PMs (v), (d)Raman spectra of dasatinib (DAS), Soluplus®, and the dasatinib-loaded Soluplus®/TPGS mixed micelles and the enlarged region of structural change, (e)Release profiles of dasatinib from dasatinib-loaded TPGS/Soluplus® mixed micelles in a PBS (pH 7.4) medium containing a 0.5 % Tween 80 medium at 37 °C, (f)In vitro study (First order).

The DSC examination of the drug showed a linear line which is an indication of the drug's excellent thermal stability. This endothermic peak of the drug at 2250 °C is sharp which means that the medication has melted. However, there is no observation of an exothermic peak for this medication. Feizi and his colleagues discovered that the condensed peak at 60.2 °C was indicative of the melting of Soluplus when it underwent a DSC analysis. This is quite evident from the formulation of the medication. The transition from a solid crystalline to a liquid crystalline state is indicated by the presence of an endothermic peak which is large [49]. As shown in Fig. 3(a), there are significant peaks at 175 °C and 225 °C in the drug formulation, while there are also visible peaks at 180 °C and 235 °C.

### 5.8 Powder X-ray diffraction (XRD)

Graphite-monochromated Cu-Kα radiation (1.5406 Å) was employed alongside an R-AXIS SPIDER diffractometer, which was equipped with an imaging plate area detector, for the acquisition of powder X-ray diffraction (PXRD) patterns at room temperature. The samples were placed on a cryoloop, and photography was conducted for a duration of 5 min while the sample underwent rotation at a pace of ten degrees per second around the φ-axis, experienced a shift of ω at a rate of one degree per second within the range of 120° and 180°, and maintained a stationary position at 45° [50]. The images were merged using the AreaMax software, with a step size of 0.05° and a span of 5°–50°. As illustrated in Fig. 3(b), the powder patterns were examined within Jade Plus to ascertain the positions and intensities of the peaks.

### 5.9 Fourier transform infrared spectrometer

The FTIR technique is considered reliable for evaluating the compatibility of all structure components included in the structural formulation - DAS, Soluplus, TPGS, a physical combination, and DAS-PMs. Through the FTIR method, the molecular interactions within the PMs' solid matrix were explored. Hydrogen bonding could potentially occur between Dasatinib and Soluplus due to their structural similarities, which might result in a shift and broadening of the FTIR absorption bands at the interacting functional groups. The possibility of hydrogen bond formation between Dasatinib aromatic OH group is shown in Fig. 3(c), which presents the FTIR spectra of pure Dasatinib, Soluplus, the physical mixture of Soluplus and Dasatinib, and DAS-PMs [51]. Distinct bands in pure dasatinib were observed, corresponding to O-H stretching at 3300 cm−1, C=O absorption at 1685 cm−1, and C=C stretching from 1500 to 1200 cm−1, consistent with previous studies. Soluplus displayed several bands, including the amide group's C=O stretch from 1625 to 1725 cm-1, the OH stretch from 3350 to 3550 cm-1, and the C-H stretch from 2923 to 2857 cm-1. TPGS also exhibited bands such as O-H at 3300 cm-1, C-H stretching from 2800 to 3000 cm-1, C=O stretching of the ester group at 1738 cm-1, and C-O stretching from 1250 to 1000 cm-1. In DAS-PMs, the carbonyl absorption peaks shifted to 1600 cm-1 and 1500 cm-1, not seen in the physical mixture, indicating possible interactions.

### 5.10 Raman spectroscopy

A Raman spectroscopy process was performed using a 532 nm laser and a Horiba Scientific microscope with a 20 × objective lens. Three different Raman spectra were captured during this process. In each spectrum, there were two peaks present; one located at about 1400 cm-1 and the other at about 4700 cm-1. The relative intensity of these peaks varied among different samples. The placebo spectrum shown in Fig. 3(d) had a weaker peak at 1400 cm-1. The DAS-PMs spectrum was found to be the closest to the "DAS" spectrum. As the quantity of DAS-PMs increased, the strength of the peak also increased. The scan range for this process was set between 1400 and 5000 cm−1. Each spectrum was the result of five scans, with each scan having an acquisition duration of 20 s. The samples were examined on an aluminium foil holder for this process. The device used for this process was calibrated using a silicon reference [52]. Temperature Differential A TMS 94 controller-equipped Linkam LTS 350 apparatus was utilized for Raman spectroscopy. A 532 nm laser was used to scan samples within a range of 100–3600 cm−1. The temperature settings were managed using Horiba Scientific 2.0 software, which facilitated the acquisition of a spectrum every 5 °C at a heating rate of 2 °C/min, up to a maximum temperature of 230 °C. The initial temperature was set at 30 °C. The plotting of all acquired spectra (20−40 acquisitions) was conducted using the Horiba Scientific software.

### 5.11 Scanning electron microscopy analysis (SEM)

**Fig. 4:**
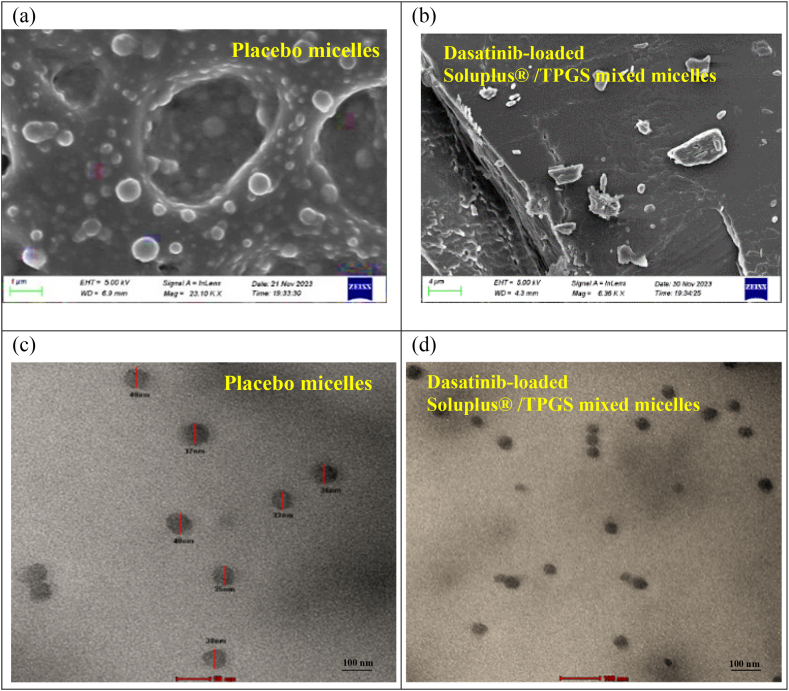
(4a) SEM image of Placebo micelles, (4b) SEM image of dasatinib-loaded Soluplus®/TPGS mixed micelles, (4c) TEM image of Placebo micelles, 4(d) TEM image of dasatinib-loaded Soluplus®/TPGS mixed micelles.

The rough edges and mono dispersion of the optimized formula were detected. Hesselink et al. used SEM to assess the surface morphology of the optimized formulas. The resulting polymeric micelles were spherical, smooth, and spherical with low aggregation and limited dispersion, as shown in the SEM images. They are naturally monodispersing. Fig. 4(a–b) [53].

### 5.12 Transmission electron microscopy analysis

The optimized formulation of the dasatinib-loaded polymeric solution demonstrated that the particles possessed a spherical morphology and maintained a uniform size of 40 nm. It was established that the formulations were consistent. A correlation was observed between the particle size data and the TEM results; the corresponding Fig. 4(c–d) presents the relevant images.

### 5.13 In vitro release

Dasatinib was in vitro freed from the Soluplus®/TPGS (2:2) mixed micelle systems and was alcohol-dissolved to form a free solution in physiological saline that contained 0.5 % Tween 80 (w/v). This is what Fig. 3(e) shows. Just 19 % of the medication was released from dasatinib-loaded mixed Soluplus®/TPGS micelles in the first 48 h [54]. Compared to dasatinib-loaded Soluplus®/TPGS mixed micelles, the dasatinib solution displayed a higher drug release rate. In a 12-h period, 19 % of the drug was released from dasatinib-loaded mixed Soluplus®/TPGS micelles, whereas 40 % of the drug was released from the dasatinib solution within the same timeframe. After 72 h, approximately 85 % of the dasatinib in the Soluplus®/TPGS (4:1) micelle systems was released from the dialysis bag. The steady incorporation of hydrophobic Dasatinib into the mixed micelle's core resulted in an enhanced sustained-release property of the Dasatinib-loaded Soluplus®/TPGS micelles. The prolonged release of Dasatinib has the potential to maintain a consistent medication concentration and extend its therapeutic benefits. Several mathematical models are utilized to quantify the drug release and its mechanism. By fitting the drug release data from DAS-PMs into several kinetic models, especially those with R^2^ values closer to 1, the straight-line equation was regarded as accurately represented. With an R^2^ value of 0.9697 in Fig. 3(f), the drug release following the assessment of the aforementioned factors was found to be of the first order.

### 5.14 In-vitro cytotoxicity studies

#### 5.14.1 MTT assay

**Fig. 5:**
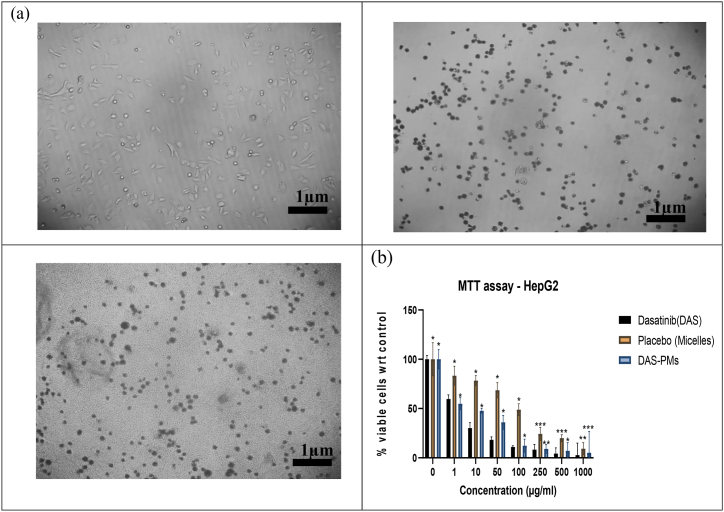
MTT Assay a) Cell inhibition percentage w.r.t control at different concentrations (0, 1, 50,100, 250, 500,1000 μM) b) Cell inhibition comparison of DAS and DAS-PMs using p-test (values are represented as mean ± SD (n = 3)) at 0.05 significance indicating (p > 0.09999: ns; p < 0.0109: ∗; p < 0.0331: ∗∗; p < 0.0001: ∗∗∗).

Fig. 5 depicts the results of the cytotoxicity test of DAS and DAS-PMs micelles, conducted via the SRB assay on HepG2 cells, in contrast to the control group. Right from the first hour of the treatment, both the pure drug and the polymeric micelles began to exhibit cytotoxic effects. In both DAS and DAS-PMs, there was a reduction in the percentage of viable cells compared to the control group. After 4 h, a significant difference between the two was observed. The experiment was conducted at intervals of 0, 1, 2, 4, 8, 12, 24, and 28 h. After 48 h, the viability of cells for DAS and DAS-PMs was found to be as significant as 12.185 ± 0.63 and 10.925 ± 0.035, respectively. This suggests that the drug was internalised and exhibited controlled activity when encapsulated in the polymeric micelles [55]. Tukey's tests were utilized for data analysis. The DAS polymeric micelles and DAS-PMs had p values of 0.0056 and 0.0026, respectively. These suggest significant results (p < 0.05). The IC-50 value of DAS-PMs was notably lower than that of DAS, indicating an increase in cytotoxicity.

#### 5.14.2 Cell cycle analysis

**Fig. 6:**
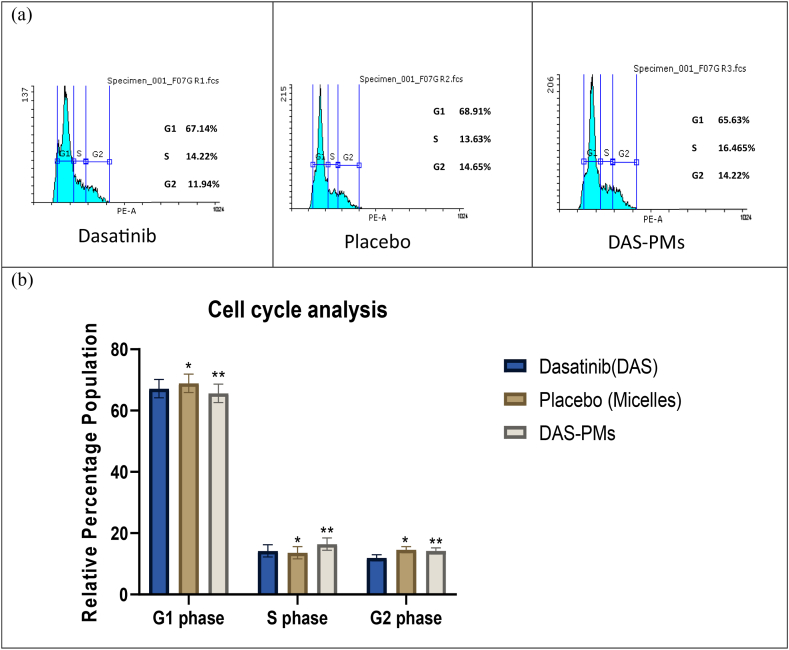
Cell cycle analysis a) Cell cycle analysis of DAS, Placebo, DAS-PMs. b) Arrest of cell cycle in HepG2 cells after treatment with DAS, Placebo, and DAS-PMs, represented as mean ± SD, showed non-significant differences (p > 0.1234), while significant differences were observed with p = 0.001 (✱✱✱) for DAS, p = 0.01 (✱✱) for Placebo, and p = 0.05 (✱) for DAS-PMs.

**Fig. 7:**
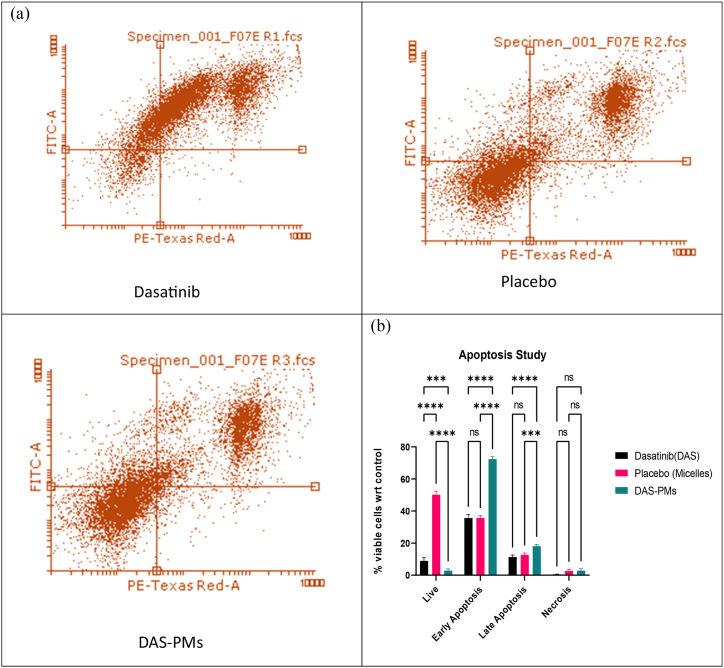
Apoptosis study a) Apoptosis analysis of DAS, Placebo, DAS-PMs. b) Apoptosis of HepG2 cell lines upon treatment with DAS, Placebo, and DAS-PMs, represented as mean ± SD, exhibited non-significant differences (p = 0.998), while significant differences were observed with p = 0.0001 (✱) for Placebo and p = 1.0004 (✱✱, ✱✱✱) for DAS and DAS-PMs.

Flow cytometry was used to analyse the effects of DAS, a placebo, and DAS-PMs Polymeric micelles on the cell cycle, following a 24-h incubation period at their individual IC50 values. The number of cells in each phase was determined by comparing the relative percentage of DNA content to the control. For DAS-PMs, the relative percentage of cells was significantly higher than for the control. According to Park et al. the formulation of polymeric micelles in the G1 and G2 phases restricts the organisation of cellular contents and reduces the metabolic activity required for cell division [56]. The medication that is pure interferes with the cell cycle's S phase, leading to apoptosis. The cell cycle distribution of the sample changed significantly due to the treatment. The treated group DAS (R1) had a higher percentage of cells in the G1 (67.14 %) and S (14.22 %) phases and a lower percentage of cells in the G2 phase (11.94 %), compared to the control group (58.18 % in G1, 20.04 % in S, and 18.14 % in G2). The Placebo (R2) treatment group had a higher percentage of cells in the G1 (68.91 %) and G2 (14.65 %) phases and a lower percentage of cells in the S phase (13.63 %). Additionally, as shown in Fig. 6, the treated group of DAS-PMs (R3) had a lower percentage of cells in the G2 phase (14.22 %) and a higher percentage of cells in the G1 (65.63 %) and S (16.45 %) phases compared to the control (see Fig. 7).

#### 5.14.3 Apoptosis study

Using flow cytometry, an apoptosis test was conducted to evaluate the importance of the outcomes of the cell reactions. This investigation elucidates the manner in which cells respond to treatment. A significant amount of apoptosis was witnessed when comparing the Dasatinib (DAS), Placebo, and DAS-PMs loaded groups to the living, early, late, and necrosis groups. A greater proportion of apoptosis was noted when compared to a medication that was not loaded with DAS-PMs [57]. The analysis's findings, as found by Liu et al. stated that after 15 min of incubation following treatment, the sample control had 63.78 % viable cells, 3.38 % early-apoptotic cells, 19.56 % late-apoptotic cells, and 13.28 % necrotic cells. The DAS (R1) results indicated that 90 % of the cells were alive, 0.6 % were in the early apoptotic phase, 72.4 % were in the late apoptotic phase, and 18.1 % were in the necrotic phase. Sample-Placebo (R2) showed 50.3 % viable cells, 2.7 % cells in the early stage of apoptosis, 35.73 % cells in the late stage of apoptosis, and 11.4 % cells in the necrotic phase. Sample-DAS-PMS (R3) displayed 48.7 % viable cells, 2.9 % early apoptotic cells, 35.7 % late apoptotic cells, and 12.7 % necrotic cells. In comparison to the control, sample-DAS (R1) had the lowest number of viable cells [58,59].

#### 5.14.4 ROS estimation with Flow cytometry – HepG2

**Fig. 8:**
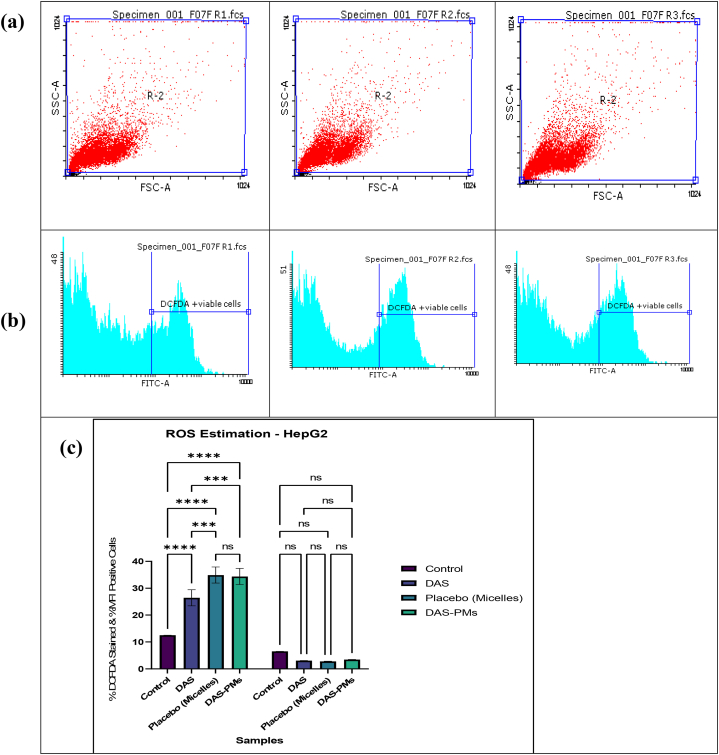
ROS estimation with flow cytometry a) ROS level analysis of Control, DAS, Placebo, DAS-PMs. b) ROS level analysis in the way of Relative % wrt control of DAS, Placebo, DAS-PMs c) Treatment of HepG2 cells with control and IC50 doses of DAS-PMs resulted in ROS levels % compared to control, with mean ± SD representation, demonstrating non-significant differences (p = 0.9849), while significant differences were observed with p = 0.0007 (✱), p = 0.0003 (✱✱), and p = 0.0009 (✱✱✱) for IC50 doses of DAS-PMs.

Sample-DAS (R1) (3.09 %), Sample-Placebo (R2) (2.82 %), and Sample-DAS-PMs (R3) (3.48 %) contained MFI-positive cells in comparison with the Control (1 %), as shown by the ROS estimation of the HepG2 cell line [60,61]. Yan et al. have stated that having high levels of reactive oxygen species (ROS) inside a cell can speed up the cell cycle being stopped which then leads to cell death. Thus, it is very important to look for ROS in HepG2 cells that have been planted with different levels of tiny bubble-like particles called polymeric micelles [62]. The purpose of the present study was to determine whether HepG2 cells generate ROS. HepG2 cells exposed to DAS-PMs exhibited significantly higher ROS production compared to the control group. This finding suggests that the use of polymeric micelles can effectively increase ROS levels, which may be attributed to their efficient cellular uptake and inhibition of cell death [63] (see Fig. 8).

#### 5.14.5 MMP estimation with Flow cytometry – HepG2

**Fig. 9:**
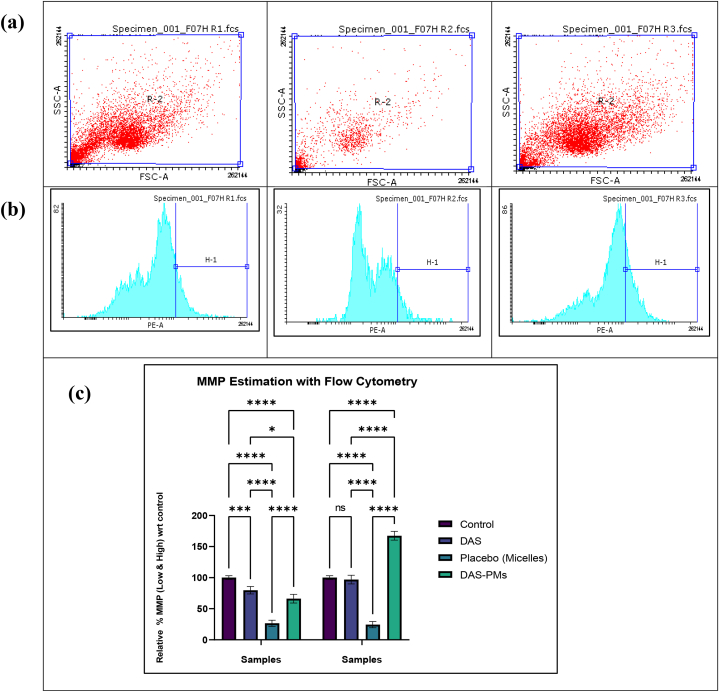
MMP estimation with flow cytometry a) Mitochondrial membrane potential change of control, DAS, Placebo, DAS-PMs b) MMP level analysis in the way of Relative % wrt control of DAS, Placebo, DAS-PMs c) MMP change, represented as relative % compared to control with mean ± SD, showed non-significant differences (p = 0.9999), while significant differences were observed with p = 0.0103 (✱), p = 0.0004 (✱✱), and p = 0.0001 (✱✱✱).

The results show that the treated sample's R3 (DAS-PMs) relative percentage was 167.363 % when the relative MMP was high, compared to the control's 100 % and the treated sample's 79.807 % when the relative MMP was low. R3 (DAS-PMs) had the highest estimated activity of all the samples [64]. On both sides of the mitochondrial membrane, protons and ions disperse unevenly, which creates the mitochondrial membrane potential, or MMP. Reduced levels of MMP could lead to the opening of transition pores, allowing the release of pro-apoptotic chemicals and mitochondrial permeability [65]. The CytC's release into the cytoplasm by the mitochondrial matrix initiates caspase-9's activation, which then initiates the mitochondrial apoptosis process. OxPhos is disrupted and MMP is reduced, leading to organelle swelling and rupture, along with ROS production [66]. The picture below shows polymeric micelles, suggesting that the cells are undergoing apoptosis or necrosis due to the disruption of their mitochondrial function, which is essential for the survival of cancer cells. The significant effect of polymeric micelles on mitochondrial function was evaluated using Tukey's multiple comparison tests, as demonstrated in Fig. 9 [67].

#### 5.14.6 In-vitro Scratch Assay

**Fig. 10:**
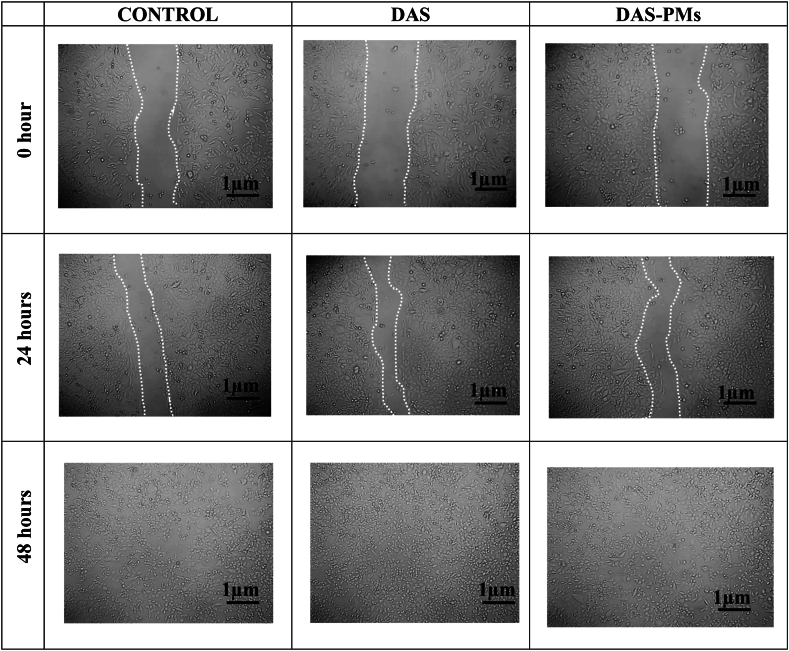
In vitro Scratch Assay: Wound healing observation of Control, DAS, DAS-PMs on 0 and 24 h and 48hrs

The aim of the in-vitro scratch test is to assess how effectively cancer cell growth is curbed. Based on the findings from the scratch wound experiment, it was observed that HepG2 liver cell lines that were treated for 24 h with control, DAS, and DAS-PMs polymeric micelles, both DAS and DAS-PMs curbed cell migration after 48 h of treatment [68]. After 48 h, the wound area in the case of polymeric micelles only slightly decreased, but in the case of pure drug therapy, cell motility can be observed, as shown in the picture below, Fig. 10.

#### 5.14.7 DNA fragmentation assay

**Fig. 11:**
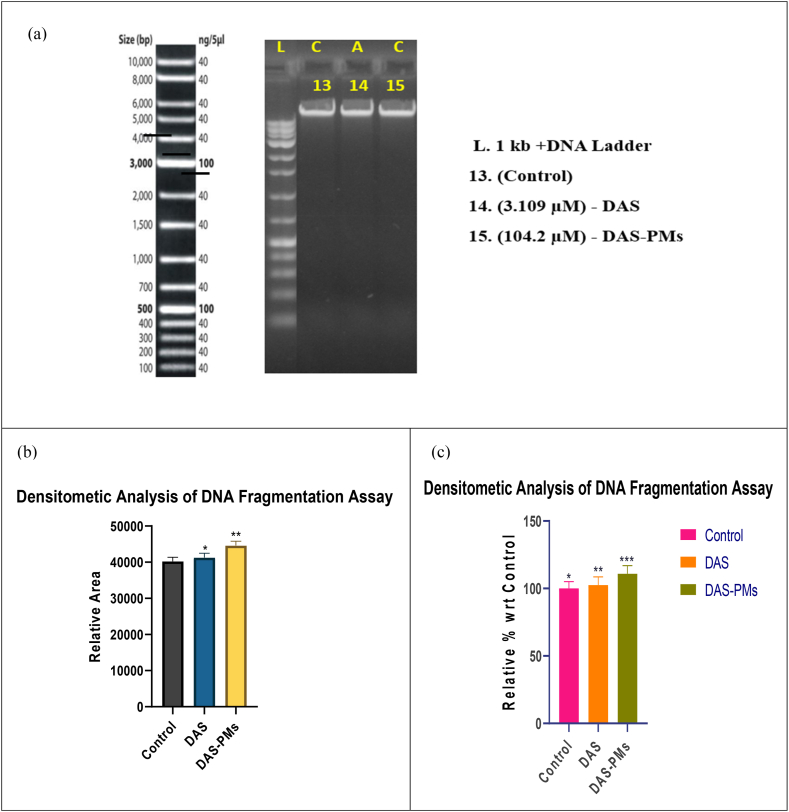
DNA fragmentation assay a) DNA fragmentation assay mentioning DNA ladder along with Control, DAS, DAS-PMs. b) Relative area of cells compared to control, DAS, and DAS-PMs using Dunet's One-way ANOVA test (mean ± SD, n = 3) showed non-significant differences (p > 0.05) for control, while DAS and DAS-PMs exhibited significance levels of p = 0.1100 (✱), p = 0.1200 (✱✱), and p = 0.1300 (✱✱✱), respectively. c) Cleavage in percent cells compared to control, DAS, and DAS-PMs using Dunet's One-way ANOVA test (mean ± SD, n = 3) revealed non-significant differences (p > 0.05) for control, while DAS and DAS-PMs exhibited significance levels of p = 0.1100 (✱✱✱), p = 0.1400 (✱✱), and p = 0.231 (✱), respectively.

After the treatment of DAS and DAS-PMs at IC50 values, HepG2 cell lines are cultured for a full day (see Fig. 11). In the DAS-PMs-treated polymeric micelle cell lines, the proportion of fragmented DNA when compared to the control is elevated at 104.2 ± 1.22 %, indicating the potential for DNA targeting as a sign of late apoptosis [69]. As demonstrated **in** Fig. 11, DNA ladders were observed in the groups treated with DAS-PMs and standard medication as evidence of apoptosis.

#### 5.14.8 Cellular uptake of DAS polymeric micelles

**Fig. 12:**
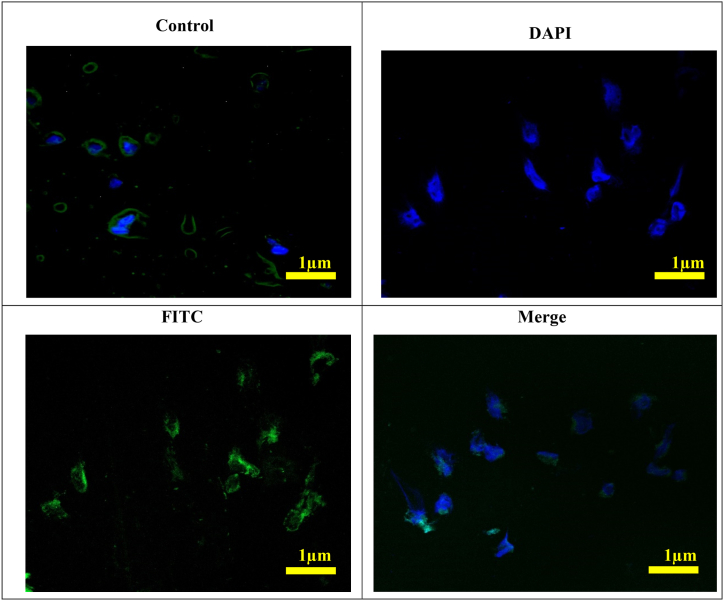
Cellular uptake of Dasatinib loaded polymeric micelles: Cellular uptake fluorescence images of samples treated with Control, DAS-PMs.

The cellular uptake of DAS polymeric micelles was evaluated by examining the uptake of DAS by HepG2, hepatocellular carcinoma, and murine melanoma cells after exposure to free DAS and DAS-PMs micelles. Cells were visualized under a fluorescence microscope, and the DAS fluorescence intensity in treated cells was measured using flow cytometry [70]. Fluorescent micrographs depicted HepG2 cells that had been treated with free DAS and DAS-PMs for durations of 0, 1, and 4 h [71]. Cells that were treated with blank micelles showed no fluorescence, as the data does not show. However, cells that were treated with free DAS at concentrations of 50 and 100 μg/mL, according to Vignesh et al. demonstrated a slight rise in fluorescence intensity at the 1 and 4 h marks. This rise was in comparison to the control group [72,73]. However, DAS-PMs may quickly accumulate in HepG2 cells, as indicated by the vivid green fluorescence—even just 1 h after. When cells were treated with DAS-PMs for 4 h, the fluorescence intensity increased much more than after 1 h. At the 1- and 4-h time points, the fluorescence intensity in cells treated with 100 μg/mL of DAS in DAS-PMs was considerably higher than that in cells treated with 50 μg/mL of DAS. The dose- and time-dependent rise in fluorescence intensity was quantitatively evaluated using flow cytometry on HepG2 cell lines, as illustrated in Fig. 12. After 4 h of incubation, the fluorescence intensity of cells treated with DAS-PMs micelles is significantly stronger than that of free DAS-treated cells [74,75].

### 5.15 In-vivo studies

**Fig. 13:**
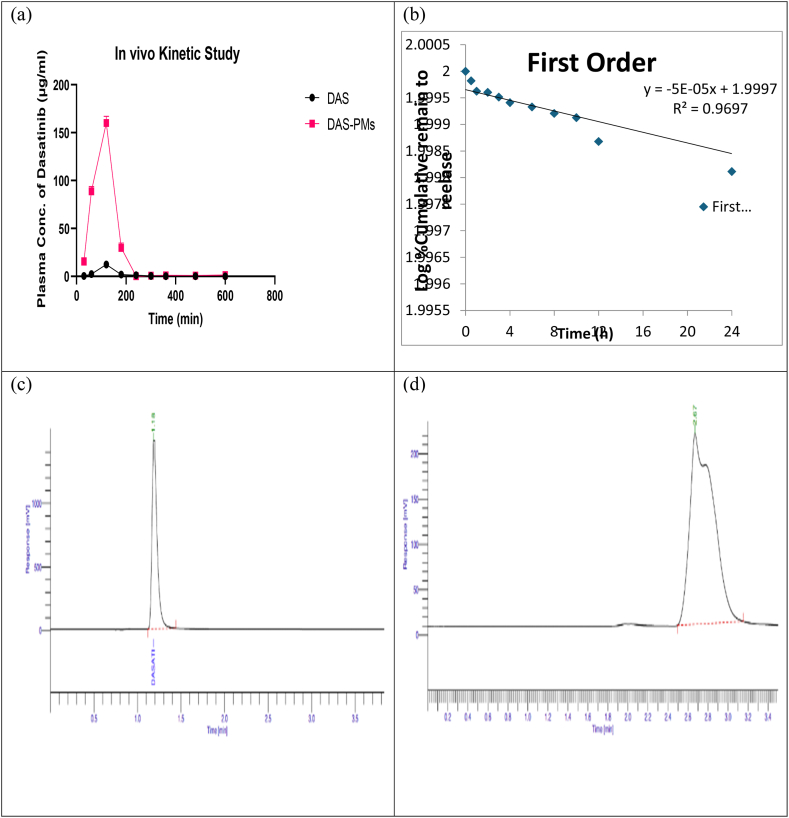
(a) In-vivo Pharmacokinetic study, (b) In-vivo HPLC peak of drug (DAS), (c) In-vivo HPLC peak of formulation (DAS-PMs).

The graph Fig. 13(a) illustrates the plasma concentration-time relationship following a solitary 5 mg/kg injection of unbound Dasatinib and Dasatinib embedded in mixed micelles. Though the rats that received mixed micelles had a reduced initial plasma concentration of dasatinib compared to those given the drug in its free form, the rats administered with mixed micelles displayed an increased dasatinib circulation time throughout the entire 72-h study (p < 0.05). The extended residence of mixed micelles was due to their prolonged circulation time and the sustained release of dasatinib [76] (see Table 6).

5 The pharmacokinetic parameters for dasatinib in plasma were evaluated by compartmental method shown in Table 7. In Fig. 13(c–d) illustrates the plasma concentration-time profiles of DAS-PMs after intravenous administration [77]. The study's results showed that the drug's Cmax and tmax, along with the formulation carrying it, were determined to be 12.33 ± 0.06 and 160.01 ± 0.07 μg/mL; 120 ± 0.0 and 120 ± 0.0 min, respectively. These results were statistically significant (p < 0.01). It was shown that mixed micelles loaded with Dasatinib can extend the elimination half-life (0.5–3) from 0.5 to 72 h (∗p < 0.01) [78]. The results showed that dasatinib-loaded mixed micelles significantly improved the in vivo bioavailability of dasatinib and extended its retention time [3,79,80]. Dasatinib-loaded TPGS/Soluplus® mixed micelles demonstrated an AUC0-∞ of 3.12275 mg/min/L, which was 2.16 times higher than that of Dasatinib alone. Additionally, the mean residual time (MRT) for the mixed micelle formulation saw a 1.3-fold increase in comparison to Dasatinib, with the difference being statistically significant (p < 0.01) [80]. Heredia et al. and White et al. suggest that their research shows that dasatinib-loaded TPGS/Soluplus® mixed micelles substantially extended systemic circulation time and reduced the rate of plasma clearance of dasatinib [81,82]. In order to measure the drug release and its mechanism, different mathematical models are used. The data of the release of drug from DAS-PMs was evaluated by fitting them into different kinetic models, the models with R^2^ values closer to 1 of the straight-line equation. After evaluating the above-mentioned parameters, the drug release followed the First order with an R^2^ value of 0.9697 in Fig. 13 (b).

**Table 6:** Analysis of variance (ANOVA) results for the responses of various prepared formulations.

Response Variable	F-value	_R_2	Adjusted R^2^	BIC	AICc
Particle size	34.11	0.9606	0.9324	25.74	36.35
Entrapment Efficacy	12.25	0.8974	0.8241	19.19	29.80

**Table 7:** Pharmacokinetic data of drug Dasatinib (DAS) and DAS-PMs.

Criterion	DAS	DAS-PMs
C_max_ (μǥ/ml)	12.33 ± 2.31	160.01 ± 2.52
T_max_ (min)	120 ± 2.48	120 ± 2.62
Volume of distribution	60.0745 ± 1.23	88.3129 ± 23.21
t_1/2_	244.632 ± 47.14	459.241 ± 52.21
AUC total (μǥ/ml)∗min	1.11188 ± 1.12	3.12275 ± 1.46
MRT (min)	198.013 ± 2.54	310.152 ± 10.21

## 6 Discussion

Liver cancer is a formidable disease characterized by its aggressive nature and limited treatment options. Despite advancements in oncology, the management of liver cancer remains challenging due to factors such as late-stage diagnosis, tumor heterogeneity, and therapeutic resistance. Dasatinib, a potent anticancer agent, holds promise for the treatment of liver cancer. However, its clinical efficacy is hindered by poor aqueous solubility and low oral bioavailability. To address these challenges, the development, optimization, and characterization of polymeric micelles for enhancing the oral bioavailability of Dasatinib represent a significant advancement in liver cancer therapy. Polymeric micelles offer a promising strategy for improving the therapeutic outcomes of anticancer drugs such as Dasatinib. By encapsulating hydrophobic drugs within a hydrophilic polymer shell, polymeric micelles enhance drug solubilization, stability, and bioavailability. This innovative approach addresses critical issues associated with conventional drug formulations, including poor aqueous solubility and limited drug absorption, particularly in the context of oral administration. Thus, the development of Dasatinib-loaded polymeric micelles represents a promising avenue for overcoming these challenges and improving treatment outcomes in liver cancer patients. The optimization of polymeric micellar formulation is crucial for ensuring effective drug delivery and therapeutic efficacy. Through a systematic optimization process, a well-defined polymeric micellar formulation was developed, tailored to enhance the oral bioavailability of Dasatinib. Characterization studies revealed a uniform size distribution of the micelles, with an average diameter of 40 nm. This optimal particle size facilitates enhanced drug solubilization and uptake, promoting efficient drug delivery to target sites. Fourier-transform infrared spectroscopy (FT-IR) confirmed the absence of chemical interactions between the drug and polymer, ensuring the integrity of the formulation. Additionally, differential scanning calorimetry (DSC) and X-ray diffraction (XRD) analyses demonstrated the amorphous nature of the drug within the micelles, enhancing its dissolution rate and bioavailability. Scanning electron microscopy (SEM) and transmission electron microscopy (TEM) further corroborated the spherical morphology and nanostructure of the micelles, essential for optimal drug encapsulation and stability. Pharmacokinetic studies elucidated a substantial improvement in the oral bioavailability of Dasatinib with the polymeric micellar formulation. Increased plasma concentration-time profiles and area under the curve (AUC) were observed compared to free drug and conventional formulations. These findings underscore the effectiveness of the micellar delivery system in overcoming barriers to drug absorption and enhancing systemic exposure. In vitro cytotoxicity assays demonstrated potent anticancer activity against liver cancer cells, with the micellar formulation exhibiting superior efficacy compared to free drug and conventional formulations. This enhanced therapeutic efficacy can be attributed to the sustained release of the drug from the micellar carrier system, facilitating prolonged exposure to tumor cells. Mechanistic studies unveiled the underlying mechanisms driving the enhanced anticancer activity of Dasatinib-loaded polymeric micelles, including the inhibition of key oncogenic pathways such as Src kinase and Bcr-Abl, induction of apoptosis, and inhibition of tumor angiogenesis. The translational potential of this innovative drug delivery system for clinical applications in liver cancer therapy is promising. Future research endeavors will focus on scaling up manufacturing processes to meet clinical demand, conducting comprehensive preclinical studies to assess safety and efficacy in vivo, and initiating clinical trials to evaluate the therapeutic benefits in patients. Furthermore, the incorporation of targeted ligands or stimuli-responsive elements into the micellar design holds potential for personalized treatment approaches tailored to individual patient profiles. The development, optimization, and characterization of polymeric micelles for oral bioavailability enhancement of Dasatinib represent a paradigm shift in liver cancer therapy. By addressing critical challenges associated with conventional drug delivery, this innovative approach offers new avenues for improving treatment outcomes and ultimately, enhancing the quality of life for patients battling this devastating disease. This detailed discussion highlights the significance, optimization process, enhanced therapeutic efficacy, translational potential, and future directions of Dasatinib-loaded polymeric micelles for liver cancer therapy, providing a comprehensive overview of the research findings and their implications.

## 7 Conclusion

The solvent evaporation approach effectively generated Dasatinib-loaded polymeric micelles (DAS-PMs) with adequate entrapment efficiency and drug loading. These micelles are characterised by their tiny particle size and low critical micelle concentration (CMC). The micelles exhibited a continuous release pattern in phosphate-buffered saline (PBS), consistent with the First-order kinetic model, suggesting that the drug release is based on Fickian diffusion. Cellular uptake experiments demonstrated a notably greater uptake of DAS-PMs in Hep G2 cell lines compared to free Dasatinib. Cytotoxicity tests confirmed that while empty micelles had no toxic effects, DAS-PMs displayed superior cytotoxicity that depended on both time and concentration, with lower IC50 values than free Dasatinib. The pharmacokinetic investigations showed that DAS-PMs had a longer duration of time in the bloodstream, which improved their effectiveness in fighting tumours. Therefore, DAS-PMs are seen as a potential drug delivery mechanism for treating hepatocellular carcinoma (HCC). However, further in vivo study is needed to confirm their therapeutic usefulness.

## CRediT authorship contribution statement

**Rehan shaikh:** Writing – review & editing, Writing – original draft, Validation, Software, Project administration, Methodology, Investigation, Data curation, Conceptualization. **Sankha Bhattacharya:** Writing – review & editing, Writing – original draft, Visualization, Validation, Supervision, Software, Resources, Project administration, Methodology, Investigation, Formal analysis, Data curation, Conceptualization. **Suprit D. Saoji:** Visualization, Validation, Supervision, Resources, Project administration, Formal analysis, Conceptualization.

## Disclosure statement

The authors declare no conflict of interest.

## Data availability

The authors confirm that the data supporting the findings of this study are available within the article references.

## Funding

The authors received no funding for this work.

## Declaration of competing interest

Authors declare there are no conflict of interest.
